# Can diode laser 810 nm decrease post endodontic pain in patients with asymptomatic necrotic maxillary incisors? A four-arm randomized controlled trial

**DOI:** 10.1038/s41405-024-00203-w

**Published:** 2024-03-14

**Authors:** Mohammad Tamer Abbara, Samar Akil, Omar Hamadah, Hassan Achour, Ghina Mahayni, Yasser Alsayed Tolibah

**Affiliations:** 1 DDs, MSc,Ph.D student at the department of Endodontics, Faculty of Dentistry, Damascus University, P.O. Box 3062, Damascus, Syria; 2 DDS,MSc,Ph.D Associate Professor at the department of Endodontics, Faculty of Dentistry, Damascus University, P.O. Box 3062, Damascus, Syria; 3 DDS,MSc,Ph.D Associate Professor at the department of Oral Medicine, Faculty of Dentistry, Damascus University, P.O. Box 3062, Damascus, Syria; 4 DDs, Faculty of Dentistry, Al-Sham Private University, P.O. Box 3062, Damascus, Syria; 5 DDs, MSc,Ph.D student at the department of Pediatric Dentistry, Faculty of Dentistry, Damascus, University, P.O. Box 3062, Damascus, Syria

**Keywords:** Diseases, Oral diseases

## Abstract

**Aim:**

To find the best method for applying the diode laser 810 nm to relieve post-endodontic pain on necrotic maxillary incisors with periapical lesions within a single-visit treatment.

**Methods:**

Eighty patients with a necrotic incisor, diagnosed with asymptomatic apical periodontitis, received standardized cleaning and shaping procedures, then divided randomly with a 1:1:1:1 allocation ratio into four groups: Group 1: control group with no laser application, Group 2: applying the diode laser as an irrigation activation system (IAS), Group 3: applying the diode laser from the buccal and palatal mucosa, Group 4: applying the diode laser as an IAS and from buccal and palatal mucosa. The postoperative pain was assessed using the visual analog scale (VAS) 1, 3, 7, and 14 days after the treatment. The mean values of the VAS score were statistically analyzed used Kruskal–Walis and Mann–Whitney U tests. The level of significance was set at a = 0.05.

**Results:**

During 14 days after treatment, there was a statistically significant difference between mean values of VAS scores in the four groups (*P* value < 0.05); Group 1 scored the highest score, whereas Group 4 showed the lowest one. Moreover, Group 4 showed favorable outcomes compared with Group 2 and Group 3 during the first three days after treatment.

**Conclusion:**

Diode laser reduced postoperative pain after necrotic teeth with large-sized apical lesion treatment, whereas using diode laser either as an IAS or LLLT reduced the postoperative pain compared with the control group. Moreover, the usage of a diode laser in both previous techniques represents the best protocol for postoperative pain relief during 14 days of treatment.

**Clinical relevance:**

The clinical significance of this study is to investigate the best method to reduce postoperative pain using diode lasers 810 nm; where the results of this study indicated that the more diode laser exposer in LLLT and IAS, the less postoperative pain after endodontic procedures.

## Introduction

Low-level laser therapy (LLLT) with diode laser incorporates the application of non-heating non-invasive red light which can penetrate biological tissue deep to 5 mm, interact with cells, and promote oxidative metabolism in the mitochondria leading to an increase in the synthesis of endogenous endorphins, modify the pain threshold, decrease bradykinin synthesis, prostaglandin synthesis, and histamine release. The aim of LLLT in dentistry is decreasing inflammation, improving tissue repair and wound healing, and producing analgesia [1–6]. Moreover, LLLT is a very useful non-invasive therapy for patients who are needle phobic or for those whom they are contraindicated to take non-steroidal anti-inflammatory drugs [7].

The diode laser has been used widely in many domains of medicine and dentistry [8]. It can be used to disinfect the root canal due to its ability to eliminate bacteria and seal the dentinal tubules [9]. In addition, diode laser can be used as LLLT, as it is more profound than the blue-visible or red-visible spectrum [10], and thus it is eligible to be applied externally to the periapical tissues to relieve pain following endodontic treatment in the mechanism mentioned previously [11, 12].

Diode lasers commonly operate at approximately 980 nm, falling within the near-infrared range of the light spectrum. This wavelength is absorbed by tissue, initiating a photobiomodulation response that can mitigate inflammation and expedite tissue recovery [13, 14].

The tissue’s response to laser therapy can be divided into two main categories: primary and secondary reactions. Primary responses entail the dilation of blood vessels (vasodilation), improved lymphatic drainage and blood circulation, heightened activity of neutrophils and fibroblasts, enhanced cellular metabolism, and an increased threshold for pain receptor stimulation. On the other hand, secondary responses involve an increase in specific prostaglandins such as PGL2, renowned for its anti-inflammatory properties, heightened production of immunoglobulins and lymphokines affecting the immune system, and augmented synthesis of beta-endorphins and enkephalins, which contribute to analgesia [15].

Moreover, diode laser can be used to agitate the irrigation solution, where it causes a vapor bubble formation which occurs very fast, causing very rapid turbulence in the solution throughout the whole canal and thus it enhances the effectiveness of cleaning process during root canal treatment [16–18].

Postoperative pain is one of the most problems facing clinicians which arises especially in the first hours or days after treating necrotic teeth with peri-apical lesions as pain and/or swelling requiring emergency clinical visit [19] and occurs with an incidence of 3–58% [20].

The inflated levels of inflammatory mediators, which exist in the damaged periapical tissue, engender the activation or sensitization of peripheral nociceptors and cause peripheral hyperalgesia [21, 22].

Many factors could be correlated with the incidence of postoperative pain included: the number of visits to complete the treatment, factors related to the host (age and gender), the existence of pre-operative pain, the condition of the pulp (vital or necrotic), and the peri-radicular tissues, and the extrusion of the debris and the medicaments beyond the apex during the treatment [23, 24].

There are many protocols to relieve the postoperative pain including pharmaceutical and non-pharmaceutical strategies. The pharmaceutical strategies aim to minimize the symptoms by using drugs such as acetaminophen [25], steroidal and non-steroidal drugs [26], anti-inflammatory [27], and a combination of 2 drugs [28]. Non-pharmaceutical strategies depend on reducing the postoperative pain with the aid of stress-lowering approaches [29], and intra-canal cryotherapy [30] and Lasers [31, 32].

Although the outcomes of using lasers in reducing the postoperative pain seems to be promising, there remains a dearth of comparative studies exploring various diode laser protocols. Questions linger regarding the relative effectiveness of diode laser as an IAS or in LLLT in mitigating postoperative pain [31], as well as whether an optimal blend of these techniques exists. Moreover, variations in parameters such as laser application duration, power settings, and specific methodologies across studies further compound the issue. Accordingly, further investigation is imperative to ascertain the most efficacious diode laser protocol for alleviating postoperative pain, so this study aimed to evaluate the effect of using the diode laser 810 nm either as an IAS to activate the triple final irrigants (Sodium Hypochlorite (NaOCl), Ethylenediamine tetraacetic acid (EDTA), and Chore hexedine (CHX)), or in LLLT by application the diode laser tip at the buccal and lingual mucosa surrounding the periapical area of the necrotic tooth, or the combination of both previous techniques on post-operative pain after endodontic treatment of necrotic teeth with apical lesions. There were two null hypotheses, the first one suggests that there are no differences between diode laser application in three ways on the post-operative pain. The second one suggests that the diode laser 810 nm has no effect on the post-operative pain compared to the control group in which no laser was used.

## Materials & methods

### Study design, settings, and ethical approval

This randomized single-blinded clinical trial (RCT) has utilized a four-arm parallel superiority design with a 1:1:1:1 allocation ratio. This study was conducted from January 2020 and January 2023 at the Endodontic Department Faculty of Dentistry, Damascus University, Damascus, Syria. This study was conducted respecting the ethical guidelines of the Declaration of Helsinki. The research project was ethically approved by Damascus University (approve number: UDDS-98-07022019/SRC-1734). The project was funded by Damascus University (funder No. 501100020595) and it was registered at the ISRCTN registry under ID number: ISRCTN99457940 in 17/11/2022. This RCT has been written according to CONSORT 2010 guidelines [33].

### Sample size calculation

The sample size was calculated using G* Power 3.1.9.4 (Heinrich-Heine-Universität, Düsseldorf, Germany). It was estimated depending on the study of Naseri et al. [11] which described the changes in a Visual Analog Scale (VAS) value after 24 h of endodontic treatment with LLLT. A minimum total sample size of 80 patients (20 in each group) was found to be sufficient for a level of significance of 0.05, power of 95%, and 0.37857 as effect size f.

### Recruitment and eligibility criteria

Two hundred seventy-five patients aged between 25 and 44 years were referred to the Endodontic Department during the study period because the presence of apical lesions in their teeth was investigated by the principal research (M.T.A). The principal investigator searched for patients with at least one or more maxillary incisors (central or lateral) with large-sized periapical lesions >5 mm (S3) according to Venskutonis classification [34]. Preoperative periapical radiographs were taken to assess incisor anatomy, periapical lesion size, apex diameter, and the cause of periapical lesion to determine the included incisors. Those who met this condition were one hundred and forty patients. Sixty patients were considered unsuitable for inclusion due to the presence of systemic diseases that compromised their general immune status, un-restorable incisors, teeth with symptomatic periodontitis, lidocaine hypersensitivity, patients with advanced periodontitis (more than 5 mm periodontal attachment and bone loss), open-apex incisors, incisors with multi-canals, internal or external resorptions, Incisors whose canals couldn’t be dried during the same treatment visit, or incisors with unsuccessful previous endodontic treatment.

Therefore, eighty patients were included in the current research. All included patients, who accepted to participate in this study, signed an informed consent sheet after explaining all the details about the trial and the therapeutic part of it.

### Randomization

Incisors were assigned to no laser application group, diode laser as an IAS group, diode laser in LLLT or the diode laser as an IAS and LLLT group together using the simple randomization method at an allocation ratio of 1:1:1:1, and a random sequence was created using the website www.random.org, which was accessed on 1 January 2020.

Thus, patients were assigned to four groups: Group 1 (control group): no laser application (*n* = 20), Group 2: diode laser as an IAS (*n* = 20), Group 3: diode laser in LLLT (*n* = 20), and Group 4: diode laser as an IAS and LLLT together (*n* = 20).

### Blinding

The present study was single-blinded; as the current study was an interventional study, the treating clinician could not be blinded regarding the diode laser application method during treatment. Moreover, the involved patients were not blinded as patients in G3 and G4 were informed that a laser would be applied to the periapical mucosal tissue. The assessment of treatment outcomes was completed by two trained researchers (two Ph.D. students) who were calibrated to the evaluation criteria and blinded to the diode laser application method.

### Clinical procedure

All clinical procedures were achieved by the principal researcher (M.T.A.). Before anesthesia, the preoperative pain was recorded using a VAS for each included incisor. Under local anesthesia (Huons Lidocaine HCL, Seoul, Korea) and rubber dam isolation with clamps number 210 (Sanctuary, Perak, Malaysia), caries and previous restorations were removed. The access cavity was refined using an Endo-Z bur (Dentsply Maillefer, Tusla, Oklahoma, USA). The canal orifice was prepared using an orifice opener file (ORODEKA LTD. Xincheng, Jining, China). The working length was determined with an apex locator (C-smart-1, COXO, Fushan, China) using K-file #10, and confirmed radially.

After achieving the glide path, the canal was shaped using Plex V ORODEKA rotary files (ORODEKA LTD. Xincheng, Jining, China). The final apical file size was (25.06) to (40.06) concerning the initial size of apex. The canal was irrigated copiously with 2 mL of 5.25% sodium hypochlorite (NaOCl) (Merck, Darmstadt, Germany) using a 30-gauge endodontic irrigating needle (Sybron Endo, Crop, Orange, CA, USA) between files. After instrumentation, the sample was divided into four groups according to the method of diode laser 810 nm application: (G1) No laser application (control group), (G2) diode laser as an IAS, (G3) diode laser in LLLT and (G4) diode laser as an IAS and LLLT together.

#### Group 1 (no laser application)

The canals were filled up with 5.25% NaOCl, and then intermittent ultrasonic irrigation was used, where a #25 U-file ultrasonic tip (U-file, Zipperer Co., Munchen, Germany), mounted on an ultrasonic handpiece (Woodpeker, Guilin, China), was inserted inside the filled canal 2 mm before the apex without wall contact. The NaOCl was activated for 45 s at the medium power setting (30-kHz) with a push-and-pull movement for each 2 mL of irrigant, which was repeated until achieving 40 mL of 5.25% NaOCl within 15 min in total. Then, all canals were irrigated with normal saline, filled with 2 mL of EDTA 17% (Produits Dentaires SA, Vevey, Switzerland), and activated with the U-file in the same previous settings for 15 s, and the procedure was repeated twice. Finally, all canals were irrigated with normal saline, filled with 2 mL of 2% CHX (Maquira, Maringa, Brazil), and activated with the U-file in the same previous settings for 15 s, and that was repeated twice. The previous method of irrigant activation was similar to the one described by Liapis et al. [35], and Prada et al. [36].

#### Group 2 (diode laser 810 nm as an IAS)

**Fig. 1:**
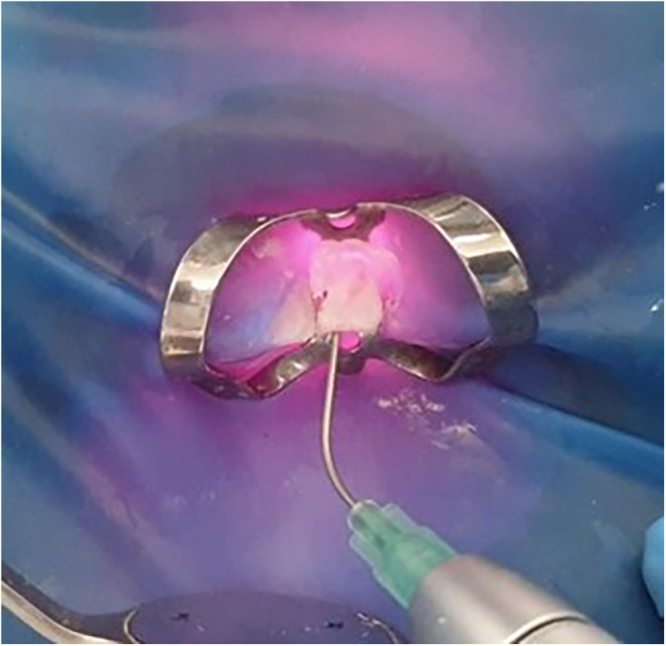
Irrigants activation using diode laser.

The canals were filled up with 5.25% NaOCl, and then the laser irradiation was performed using an 810-nm diode laser (Mercury G10; Wuhan Pioon Technology Co., Ltd., Wuhan City, China) using an optical fiber tip (200 µm). The tip was inserted 2 mm before the apex and operated at the following settings: a peak power of 2.4 W, an average power of 1.2 W, a lower frequency of 50 Hz, a 50% duty cycle, an energy of 12 J (each cycle), and in pulsed mode (5 m.s). Then, the tip was removed using a slow helical movement in the apicoronal direction. Each 2 mL was activated for 45 s and repeat until 40 mL of 5.25% NaOCl was activated within about 15 min in total (Fig. 1). Then, all canals were irrigated with normal saline, filled with 2 mL of EDTA 17%, and activated with a diode laser in the same previous settings for 15 s, and the procedure was repeated twice. Finally, all canals were irrigated with normal saline, filled with 2 mL of CHX 2%, and activated with a diode laser for 15 s, and that was repeated twice. The previous method of irrigant activation with a diode laser was similar to the one described by Coelho et al. [37] and Kaplan et al. [38].

**Fig. 2:**
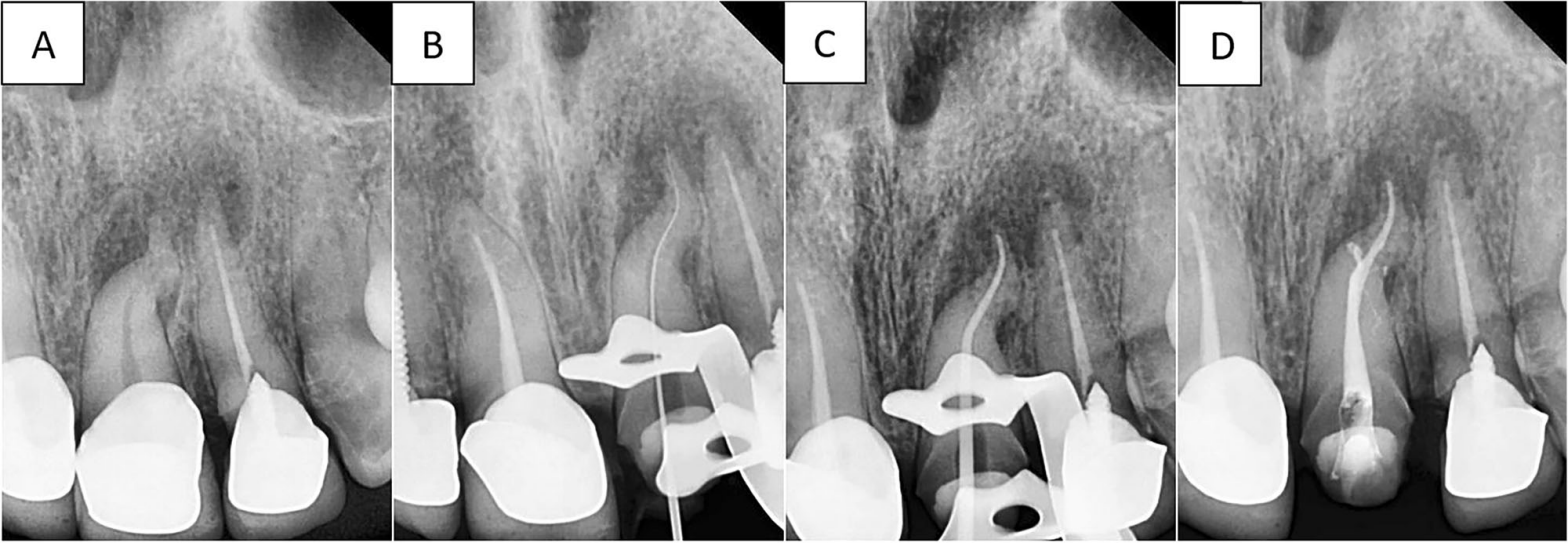
Endodontic procedure of maxillary necrotic central incisor in G1. Steps of the provided endodontic treatment in patients of G1 (**A**) diagnostic periapical radiograph, then (**B**) Working length determination, then (**C**) Cone Fit, and Finally (**D**) Final obturation.

**Fig. 3:**
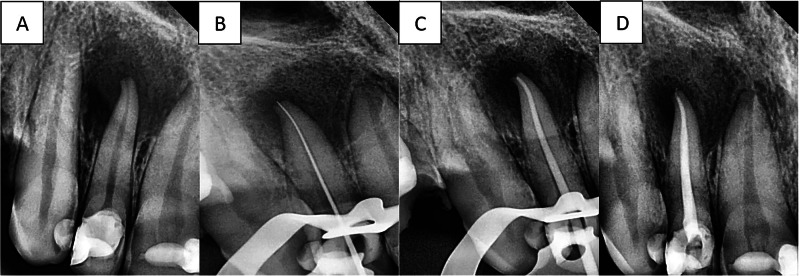
Endodontic procedure of maxillary necrotic central incisor in G2. Steps of the provided endodontic treatment in patients of G2 (**A**) diagnostic periapical radiograph, then (**B**) Working length determination, then (**C**) Cone Fit, and Finally (**D**) Final obturation.

All canals in G1 and G2 were dried using sterilized paper points (Gabadent, Guangdong, China) and obturated using gutta-percha (Gabadent, Guangdong, China) and AH plus sealer (Dentsply Sirona, Charlotte, NC, USA) in the continuous vertical waves, and the canals were restored with suitable resin bonded restorations (Figs. 2 and 3).

#### Group 3 (diode laser 810 nm in LLLT)

**Fig. 4:**
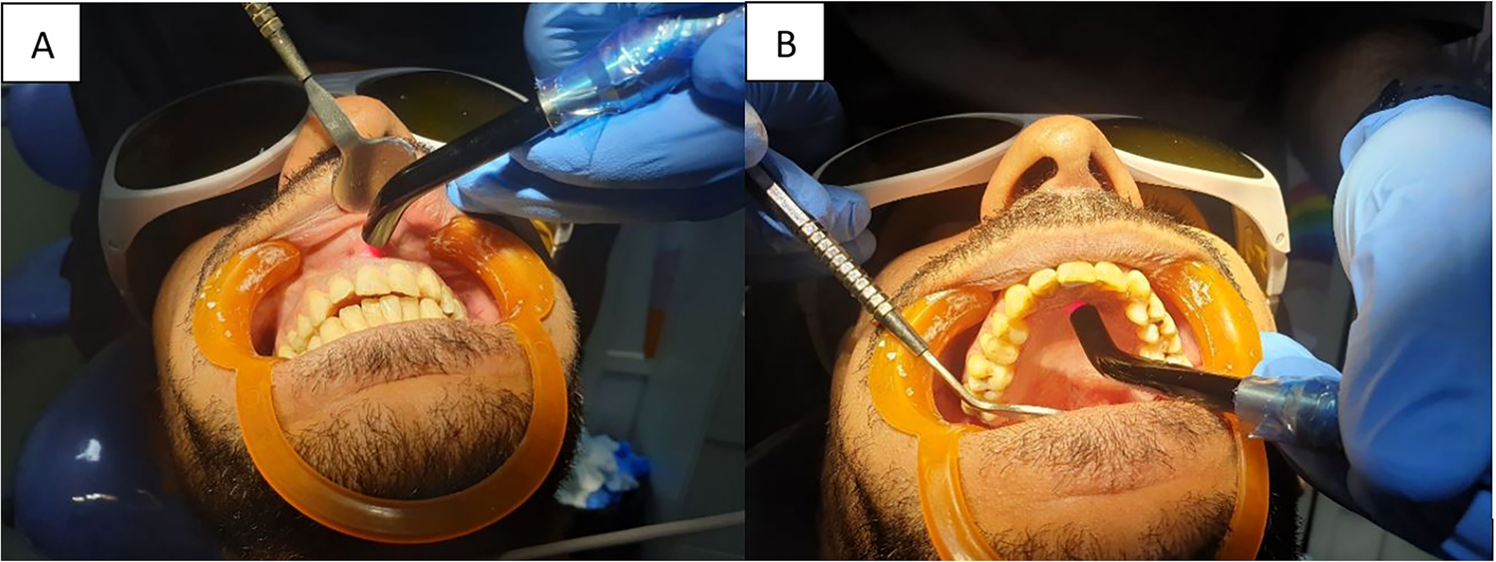
Low-level laser therapy procedures in G3 and G4. Appling the diode laser in low-level laser therapy. **A** Buccal application, and (**B**) Palatal application.

Patients in G3 received similar procedures as described in G1. However, after restoring the teeth, an 810-nm diode laser (Mercury G10; Wuhan Pioon Technology Co., Ltd., Wuhan City, China) coupled with an optical fiber laser tip (8 mm) was positioned 3 mm away from the oral mucosa and perpendicular to soft tissue with the beam focused on the apical location of the targeted tooth which was estimated based on the working length of the incisor. Diode laser exposure was carried out using a continuous mode with 10 Hz frequency, and power of 100 mW. The tip was applied both at the buccal and the palatal side for 80 s each (Fig. 4). The previous method of LLLT was similar to the one described by Naseri et al. [11].

#### Group 4 (diode laser 810 nm as an IAS and LLLT together)

Patients in G4 received similar procedures as described in G2, However, after restoring the teeth, they get LLLT with diode laser as described in G3.

### Outcomes measures

Patients of all groups were recalled at 1 day, 3 days, 7 days, and 14 days of treatment, and postoperative pain was recorded using a VAS scale, where patients chose their pain levels by pointing along a 10-cm continuous line between two endpoints, ranging from the absence of pain to unbearable pain. Moreover, participating patients were asked to report if any type of analgesic was taken, its dose and frequency.

**Fig. 5:**
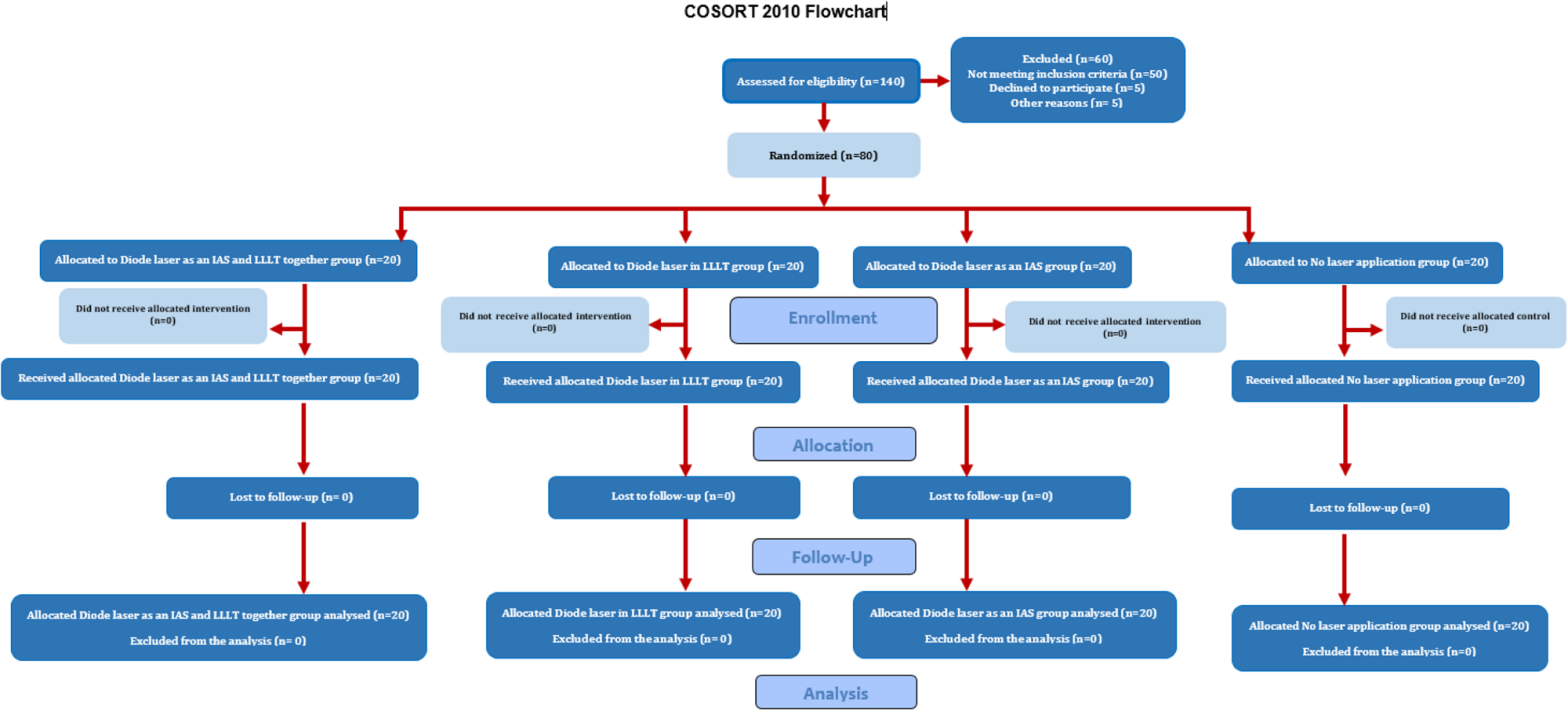
The flow chart of the study.

The CONSORT flow chart of the study was described in Fig. 5.

### Statistical analysis

The collected data were tabulated and analyzed using SPSS software (Version 20, IBM SPSS Inc., Chicago, IL, USA). Shapiro–Wilk test was used to evaluating if the quantitative measurements showed normal distribution, so the comparison between groups regarding postoperative pain was performed using the Kruskal–Wallis test, and the pairwise comparisons were performed using the Mann–Whitney U test. The level of significance was set at 0.05.

## Results

A total of 80 necrotic incisors in 80 patients (36 males and 44 females) aged between 25 and 44 years ($\bar{x}$ = 32.61) were included in the study. No significant differences were reported between the groups regarding the age (*P* = 0.814) and gender (*P* = 0.257) of the treated patient, indicating that the allocation of patients into study groups was randomized. No patient reported taking any kind of analgesics during the follow-up periods. Age and gender distributions across groups and statistical tests’ results for their comparison among groups are presented in Tables 1 and 2 respectively.

**Table 1:** Descriptive and analytic statistics of age distribution across groups.

Method of using diode laser	Mean	Standard deviation	Minimum	Maximum	*P* value^a^
No laser application	32.75	4.789	27	44	0.814
Diode laser as IAS	31.90	5.088	26	42	
Diode laser in LLLT	33.45	5.434	25	43	
Diode laser as IAS and LLLT together	32.35	5.566	25	43	

^a^One-Way ANOVA.

**Table 2:** Descriptive and analytic statistics of gender distribution across groups.

Method of using diode laser	Sex		Total	*P* value^a^
	Male	Female		
No laser application	10 (50%)	10 (50%)	20 (100%)	0.257
Diode laser as IAS	12 (60%)	8 (40%)	20 (100%)	
Diode laser in LLLT	8 (40%)	12 (60%)	20 (100%)	
Diode laser as IAS and LLLT together	6 (30%)	14 (70%)	20 (100%)	
Total	36 (45%)	44 (55%)	80 (100%)	

^a^Chi-square test.

Table 3 summarizes the mean, the standard deviation, the range, and the Kruskal–Wallis test results of the VAS scores of the pre-operative pain and the postoperative pain after 1, 3, 7, and 14 days of treatment in the groups. The Kruskal–Wallis test showed a significant difference between the groups (No laser application, diode laser as an IAS, diode laser in LLLT and diode laser as an IAS and LLLT together) after 1, 3, 7, and 14 days of treatment (*P* = 0.000, *P* = 0.000, *P* = 0.004, and *P* = 0.038 respectively). Meanwhile, there were no significant differences between the groups before the treatment (*P* = 0.778), indicating that pre-operative pain levels of the asymptomatic patients were similar.

**Table 3:** Descriptive statistics of the VAS scores in groups and the *P*-values of significance testing.

Studied period	Method of using diode laser	Number	Mean ± Std. deviation	Range	Chi square	*P* value^a^
Before Treatment	No laser application	20	0.50 ± 0.52	0–1	1.097	0.778
	Diode laser as IAS	20	0.70 ± 0.48	0–1		
	Diode laser in LLLT	20	0.60 ± 0.51	0–1		
	Diode laser as IAS and LLLT together	20	0.50 ± 0.52	0–1		
After 1 day	No laser application	20	5.00 ± 2.05	3–9	31.719	0.000^b^
	Diode laser as IAS	20	3.20 ± 1.61	2–6		
	Diode laser in LLLT	20	0.80 ± 0.63	0–2		
	Diode laser as IAS and LLLT together	20	0.20 ± 0.42	0–1		
After 3 days	No laser application	20	2.80 ± 1.68	1–6	29.349	0.000^b^
	Diode laser as IAS	20	1.60 ± 0.96	1–4		
	Diode laser in LLLT	20	0.40 ± 0.51	0–1		
	Diode laser as IAS and LLLT together	20	0 ± 0	0–0		
After 7 days	No laser application	20	0.80 ± 0.91	0–3	13.519	0.004^b^
	Diode laser as IAS	20	0.40 ± 0.69	0–2		
	Diode laser in LLLT	20	0 ± 0	0–0		
	Diode laser as IAS and LLLT together	20	0 ± 0	0–0		
After 14 days	No laser application	20	0.40 ± 0.51	0–1	8.412	0.038^b^
	Diode laser as IAS	20	0.20 ± 0.42	0–1		
	Diode laser in LLLT	20	0 ± 0	0–0		
	Diode laser as IAS and LLLT together	20	0 ± 0	0–0		

^a^Kruskal–Wallis test.

^b^Significant differences.

The Mann–Whitney U test was used to detect differences in pairwise comparisons in Table 4, where it showed that after one day of the treatment, the four groups have significant differences in pairwise comparisons (*P* < 0.05 in each pairwise comparisons); the no laser application group (G1) had the highest mean of VAS scores ($\bar{x}$ = 5.00), then the diode laser as an IAS group (G2) ($\bar{x}$ = 3.20), then the diode laser in LLLT group (G3) ($\bar{x}$ = 0.80), and the diode laser as an IAS and LLLT together (G4) had the lowest mean of VAS scores ($\bar{x}$ = 0.20). Similarly, after 3 days of treatment, the four groups have significant differences in pairwise comparisons (*P* < 0.05 in each pairwise comparisons) except that when the no laser application group was compared to the diode laser as an IAS group (*P* = 0.072); the no laser application group (G1) had the highest mean of VAS scores ($\bar{x}$ = 2.80), then the diode laser as an IAS group (G2) ($\bar{x}$ = 1.60), then the diode laser in LLLT group (G3) ($\bar{x}$ = 0.40), and the diode laser as an IAS and LLLT together (G4) had the lowest mean of VAS scores ($\bar{x}$ = 0.00).

**Table 4:** Descriptive statistics of pairwise comparisons between the groups, the *P*-values of significance testing and confidence intervals.

Studied period	Irrigant activation technique (I)	Irrigant activation technique (J)	Mann–Whitney U value	*P*-value^a^	95% CI^b^		Median^c^
					Lower	Upper	
After 1 day	Diode laser as IAS	No laser application	24.5	0.048^d^	0	3	2
		Diode laser as an IAS and LLLT together	0	0.000^d^	2	5	2
		Diode laser in LLLT	3	0.000^d^	1	4	2
	Diode laser as an IAS and LLLT together	No laser application	0	0.000^d^	3	6	4
		Diode laser in LLLT	24	0.025^d^	0	1	1
	Diode laser in LLLT	No laser application	0	0.000^d^	3	6	4
After 3 days	Diode laser as IAS	No laser application	27.5	0.072	0	2	1
		Diode laser as an IAS and LLLT together	0	0.000^d^	1	2	1
		Diode laser in LLLT	12	0.002^d^	0	2	1
	Diode laser as an IAS and LLLT together	No laser application	0	0.000^d^	1	3	3
		Diode laser in LLLT	30	0.029^d^	0	1	0
	Diode laser in LLLT	No laser application	6	0.001^d^	1	3	2
After 7 days	Diode laser as IAS	No laser application	36	0.235	0	1	0
		Diode laser as an IAS and LLLT together	35	0.068	0	1	0
		Diode laser in LLLT	35	0.068	0	1	0
	Diode laser as an IAS and LLLT together	No laser application	20	0.005^d^	0	1	1
		Diode laser in LLLT	50	1.000	0	0	0
	Diode laser in LLLT	No laser application	20	0.005^d^	0	1	1
After 14 days	Diode laser as IAS	No laser application	40	0.342	0	1	0
		Diode laser as an IAS and LLLT together	40	0.146	0	0	0
		Diode laser in LLLT	40	0.146	0	0	0
	Diode laser as an IAS and LLLT together	No laser application	30	0.029^d^	0	1	0
		Diode laser in LLLT	50	1.000	0	0	0
	Diode laser in LLLT	No laser application	30	0.029^d^	0	1	0

^a^Mann–Whitney U test.

^b^CI: Confidence interval for the difference between the group’s medians using the Hodges–Lehmann estimation.

^c^Hodges–Lehmann median.

^d^Significant differences.

After 7 days of treatment, there were no significant differences between diode laser as an IAS group (G2) ($\bar{x}$ = 0.40), diode laser in LLLT group (G3) ($\bar{x}$ = 0.00), and the diode laser as an IAS and LLLT together (G4) ($\bar{x}$ = 0.00) (*P* > 0.05, in each pairwise comparisons) regarding the mean of VAS score. However, the no laser application group (G1) had the highest mean value of VAS score ($\bar{x}$ = 0.80), and there were significant differences between G1 and each of G3 and G4 (*P* = 0.005, in each pairwise comparisons). Similarly, After 14 days of treatment, there were no significant differences between diode laser as an IAS group (G2) ($\bar{x}$ = 0.20), diode laser in LLLT group (G3) ($\bar{x}$ = 0.00), and the diode laser as an IAS and LLLT together (G4) ($\bar{x}$ = 0.00) (*P* > 0.05, in each pairwise comparisons) regarding the mean of VAS score. However, the no laser application group (G1) had the highest mean value of VAS score ($\bar{x}$ = 0.40), and there were significant differences between G1 and each of G3 and G4 (*P* = 0.029, in each pairwise comparisons).

## Discussion

The current study had special importance, although the last systematic review recommended conducting more studies on the LLLT [12], this study evaluated the effect of both intra-canal and extra-canal diode laser 810 nm application with standardized parameters in comparison with conventional endodontic treatment without the use of laser in the term of postoperative pain in single-rooted teeth presenting with necrotic pulps and periapical lesion performed in a single visit within a subgroup of Syrian patients. Our results reject the two null hypothesizes, as the three ways of diode laser application (IAS, LLLT, and combination of IAS and LLLT) reduced the postoperative pain in comparison with the control group (no laser application) during 14 days of the treatment of necrotic maxillary incisors in single visit treatment. Moreover, the three ways of the diode laser 810 nm application had different effects on the postoperative pain.

Although the rotary preparation systems reduce the extrusion of the debris beyond the apex, they do not eliminate this phenomenon [39], which increases the postoperative pain especially in necrotic teeth, where the microorganisms within the root canal system are expelled with the debris into the periapical tissues [16, 40]. This may explain the use of different methods to reduce postoperative pain after necrotic teeth treatment.

Previous studies used a diode laser in wavelength between 808–910 nm as a non-invasive way to reduce postoperative pain [3]. A lot of studies have focused on the use of diode lasers in activating irrigants as a potential method for reducing the postoperative pain [32, 41, 42], other studies have concentrated on its use in the context of low-level laser therapy [11, 43], and one study has compared both of these techniques on the postoperative pain [31], but to the best of our knowledge, this is the first study to compare the use of these two techniques together on the postoperative pain.

This study was carried out on healthy participants having asymptomatic periodontitis in maxillary incisors with a large-sized periapical lesion. Patients having a previous history of pain were not involved to reduce all potential pre-operative factors. Moreover, patients with disseminated oral pain were not included, as pain in one tooth may affect other teeth [44]. In the current study, p‐values for age and gender showed that the allocation of patients into various groups was randomized, which means that patient sample had similar distributions among the three groups. Therefore, the effect of these variables was ignored.

A specific methodology was used in the present study to minimize the postoperative pain as much as possible, where all canals were kept gently patent with a small instrument (#10 K file), and the WL was determined with the apex locator and confirmed by a radiograph to be 1 mm before the radiographic apex to avoid further periodontal ligament damage. Moreover, the canals were prepared as appropriate for the initial apex size measurement, and care was taken to avoid over-instrumentation of the root canal system to avoid increasing the postoperative pain in necrotic teeth [45]. In addition, irrigation protocols are very contrasting through in-vivo studies due to many available irrigation procedures and tooth statutes [46]. For example, the activation time, NaOCl concentration, and total volume were increased to make the irrigation protocol suitable for the treatment of necrotic teeth with large-sized periapical lesions to minimize the bacteria virulence in the root canal system. Nevertheless, continuous replenishment during the activation of the three irrigants was done to maintain their efficacy [47, 48].

The PUI was adopted in the control group because this method is commonly and widely used as an IAS in laboratory, bacteriological, and clinical studies related to endodontics [49–51]. It was also mentioned that PUI can reduce the postoperative pain compared to conventional irrigation [52].

A VAS was used in this study to evaluate the post-operative pain before and after the endodontic procedures because it is an easy numerical rating scale that has high reliability in acute pain assessments [53].

It is noteworthy that both PUI and diode laser activation didn’t increase the apical extrusion of irrigants in comparison with conventional needle irrigation [54]. Diode Laser activation of hypochlorite enhances biofilm removal from the infected dentinal root and helps to eliminate enterococci faecalis by 98% [55, 56]. It is also proposed that intracanal laser activation may eradicate microorganisms present past the root apex [57]. Nevertheless, diode Laser activation of EDTA enhances removing the smear layer from the apical third of the root canal [58]. Moreover, diode laser activation of CHX increased its penetration through dentinal tubes [59]. The previous enhancement in irrigants features, which increased in disinfecting and cleaning of the root canal system rather than the healing characters of the diode laser itself [60], may reflect in pain decreasing in patients of diode laser as an IAS group in comparison with the control group through all follow-up periods.

The previous results are in agreement with the study of Erkan and colleagues, where they found that laser stimulation led to significantly lower VAS scores than ultrasonic stimulation two and seven days of treatment [32]. However, Omar and colleagues [41] and Simpson and colleagues [42] found that the diode laser did not reduce the post operative pain in comparison with other activation methods. The reason may be due to the fact that in the study of Omar and colleagues used a diode laser 980 nm in comparison with ultrasonic activation on necrotic anterior teeth and premolars. Additionally, they did not specify the condition of the periapical tissues, particularly whether there was a periapical lesion present or not. Moreover, the study of Simpson and colleagues used a diode laser 980 nm in comparison with sonic activation on symptomatic periodontitis molars.

The application of diode laser in the LLLT (third and fourth groups) was adopted to be irradiated on the buccal and palatal surface because it represented the best protocol for this type of treatment compared to the buccal application alone during the first hours after the end of treatment, as irradiating on only the buccal surface, the periapical region close to the palatal surface may receive lower energies than the periapical region close to the buccal surface and vice versa [11].

The results indicated that the LLLT group showed improvement in mean values of VAS scores compared with the no laser application group through follow-up periods. These results met the previous studies that using a diode laser in LLLT reduced the postoperative pain [11, 43]. These results are emphasised by the fact that LLLT inhibits the production of pro-inflammatory factors and pain-related neurotransmitters [61], while also promoting the elimination of pain-inducing substances such as histamine, substance P, and dopamine [62]. These biological activities provide a plausible explanation for the positive outcomes observed in this study.

The application of a diode laser in LLLT showed superior improvement to the mean value of VAS scores in the IAS group during the first 3 days of treatment. The previous result can be explained by the diameter of the optic fiber of the device used (200 µm for the IAS group and 8 mm for the LLLT group), as the application of the laser in LLLT included a wider area than its application within the canal. The previous result differed from Ismail’s study, where the previous study found that the application of the diode laser in LLLT was better than its application as an IAS only on the first day of treatment [31]. The reason may attribute to the fact that the study of Ismail and colleagues included symptomatic and asymptomatic cases and using diode laser 910 nm.

According to the current results, the diode laser as an IAS and LLLT together group showed the lowest postoperative pain mean values compared to the LLLT group alone and IAS group alone through three days of treatment, and compared to the no laser application group through fourteen days of treatment. This result may be explained by the hypothesis that the greater the area exposed to various diode laser methods, the more effective the reduction of postoperative pain. Additionally, the combination of the two previous described effects of both IAS and LLLT at the same time led to better results.

Although both forms of intra-canal and extra-canal laser application have the same effect in relieving the postoperative pain after 3 days of treatment, the intra-canal application seems to be easier because it also activates the irrigants and thus shortens the treatment steps.

Despite our best attempts to standardize the criteria of the patients included in the current study, there were problems represented in the pain of the infiltration anesthesia and gingival pain resulting from the rubber dam clamp, which might affect pain assessment, especially on the next day after treatment. Moreover, since the previous study was a clinical study, it was not possible to standardize the apical diameters of the teeth included in the patients, so we only relied on expanding the apical foramen to three measurements of its basic diameter, and therefore the amount of expansion was not associated with the postoperative pain severity. In addition, not all cases of asymptomatic necrotic incisors were suitable for single-visit treatment, as some cases were not obturated in the same visit because the canals were not dried; these cases required a calcium hydroxide dressing that could mask the pain caused by irrigants activation [38], which forced us to exclude these cases from the current study.

Therefore, with numerous cases excluded, the opportunity to incorporate a control group utilizing a placebo or mock laser to explore psychological effects was precluded.

A further limitation is the subjective nature of the VAS, although the VAS has been used extensively in studies assessing postoperative pain, objective measurements like the initial periapical lesion size of the samples could be add.

Further studies with double-blinded, controlled with placebo, and with different pulp and periapical statues are required to prove the diode laser efficacy in postoperative pain relief. Moreover, it is mandatory to assess the relationship between the diode laser application method used and periapical lesion healing of necrotic teeth.

## Conclusion

Within the above-mentioned limitations of this randomized clinical trial, the results showed that diode laser 810 nm can reduce the postoperative pain of necrotic teeth with large-sized apical lesions; diode laser as an IAS has favorable effects in reducing postoperative pain compared to no laser application during three days of treatment. Moreover, diode laser in LLLT has favorable effects in reducing postoperative pain compared to IAS during three days of treatment, and the usage of both techniques together represents the best protocol for postoperative pain relief during fourteen days of treatment.

## Data Availability

Data can be obtained by the corresponding author upon request.
